# Exploring the experiences and priorities of women with a diagnosis of ovarian cancer

**DOI:** 10.1007/s00520-023-07903-3

**Published:** 2023-06-30

**Authors:** Maree Pasvanis, Sue Hegarty, Hayley Russell, Michelle Peate, Jennifer L. Marino

**Affiliations:** 1 Department of Obstetrics and Gynaecology, Royal Women’s Hospital, University of Melbourne, Parkville, VIC Australia; 2 Ovarian Cancer Australia, Melbourne, VIC Australia; 3 Centre for Adolescent Health, Murdoch Children’s Research Institute, Parkville, VIC Australia; 4 Department of Paediatrics, University of Melbourne, Parkville, VIC Australia; 5 Centre for Epidemiology and Biostatistics, University of Melbourne, Parkville, VIC Australia; 6 Discipline of Child and Adolescent Health, University of Sydney, Westmead, NSW Australia

**Keywords:** support resources, care resources, priority ranking, support services

## Abstract

**Purpose:**

Ovarian cancer is the third most common gynaecological cancer among women, yet remains under-researched. Past studies suggest that women who present with ovarian cancer have more supportive care needs compared to women experiencing other gynaecological cancers. This study explores the experiences and priorities of women with a diagnosis of ovarian cancer and whether age may influence these needs and experiences.

**Methods:**

Participants were recruited by a community organization, Ovarian Cancer Australia (OCA), via a social media campaign promoted on Facebook. Participants were asked to rank priorities around living with ovarian cancer, and to endorse which supports and resources they had used to address those priorities. Distributions of priority rankings and resource use were compared by age (19-49 vs. 50+ years).

**Results:**

Two hundred and eighty-eight people completed the consumer survey and most respondents were 60-69 years (33.7%). Priorities did not vary by age. Fear of cancer recurrence was identified by 51% respondents as the most challenging aspect of having ovarian cancer. Compared with older respondents, a higher proportion of young participants were more inclined to use a mobile app version of the OCA resilience kit (25.8% vs 45.1%, *p*=0.002) and expressed interest in using a fertility preservation decision aid (2.4% vs 25%, *p*<0.001).

**Conclusion:**

Fear of recurrence was participants’ primary concern, presenting an opportunity to develop interventions. Information delivery needs to consider age-specific preferences to better reach the target audience. Fertility is more important to younger women and a fertility preservation decision aid may address this need.

## Introduction

Ovarian cancer is the third most common gynaecological cancer, yet remains under-researched [1, 2]. There are multiple treatment options that are considered first-line care for treating ovarian cancer, but despite this, recurrence occurs in as many as 70% of cases [3]. The remittent cycle of recurrence and poor prognosis distinguishes women with ovarian cancer from other cancer patients [4].

Supportive care needs of women experiencing ovarian cancer vary. The most frequently reported needs fall into psychological and system/information domains, mainly fear of cancer progression/recurrence, and treatment concerns – particularly adverse side effects, and options related to future parenthood and family planning [5]. Needs may conflict, and supportive care resources are finite. Identifying patient priorities, a central tenet of patient-centred care, addresses both these issues.

Priorities may vary over time as women’s experiences with cancer change. Priorities may also vary by age. For instance, as definitive treatment for ovarian or other gynaecological cancers typically requires bilateral salpingo-oophorectomy and hysterectomy, treatment-related infertility and fertility preservation is of particular interest to women diagnosed before 50 years of age [6, 7]. Other priorities which may also be influenced by age are the delivery of health information and interaction with technology [8]. While a growing proportion of older women is accessing health information technologies, an age divide remains. Studies have shown that few health information websites have been designed to accommodate a wide audience; they typically target a younger audience [9, 10]. This could explain why many older people are dissatisfied with the health information they are able to find online [11]. It is true of most cancers, ovarian cancer included, that the majority of people who are affected are older people [12]. Therefore, it is important for us to be able to cater to the priorities of women whatever their age by providing them with tailored online health information. As patient experience data has traditionally been limited, it has been difficult to provide the level of patient-centred care that these women require and which can be used to offer them better patient-centred care and ultimately progress toward improving their quality of life.

Consumer support organisation Ovarian Cancer Australia (OCA) conducted a survey in 2017 to assess the psychosocial and informational needs of patients and survivors of ovarian cancer. Using these data, the present study aimed to explore the experiences and priorities of women with ovarian cancer and whether these priorities differed among women according to age.

## Methods

### Ethics and consent

This is a secondary analysis of data collected jointly by OCA and a commercial consultancy (Healthcare Management Advisors Pty) to guide the delivery of support services and information for women living with ovarian cancer [13]. As the purpose of original data collection was not research, Human Research Ethics Committee oversight and informed consent to research were not appropriate. Data were collected anonymously by web survey and respondents could end the survey at any time, limiting risk to participants. Although respondents were offered the option to provide contact information at the end of the survey, all such identifiers were removed from the dataset furnished by OCA for secondary analysis. Approval for the secondary analysis was provided by the University of Melbourne Human Research Ethics STEMM2 Committee, under reference number 2022-24679-35230-3. This analysis was conducted in accordance with the guidelines of the University of Melbourne, the National Health and Medical Research Council (NHMRC), and the Declaration of Helsinki.

### Recruitment

Participants were recruited by OCA though a 5-week Facebook social media campaign. An estimated 1500 people were “reached” (were shown material related to the campaign), of whom 288 completed the survey.

### Data collection and analysis

Demographics details collected included age (in ten-year categories from 19 to 89), gender (female, male, prefer not to specify), and locality remoteness using the Accessibility/Remoteness Index of Australia, derived from postcodes [14] (major city, inner regional, outer regional/remote). Clinical details included cancer stage at diagnosis, current status (diagnosis, primary treatment, post-treatment, recurrence, remission, end-of-life/palliation), and time since first diagnosis. Participants ranked nine aspects of living with ovarian cancer from 1 (most) to 9 (least) challenging (“priority rankings”): fear of cancer recurrence, feeling isolated, financial concerns, finding information on treatment options, body image issues, finding support for family and friends, changes to sexual function, finding information on genetic counselling and travelling costs. Participants also selected which support and information resources they had used to address these priorities. Resources included: friends and family; cancer support group; online forum/community; telephone service or group; one-on-one counselling; hospital treatment team; OCA website; other websites; webinars or podcasts; information sessions; other hospital services such as social work or psychology; and “other – please specify”. Participants were asked which support and information resources from OCA they had ever used: resilience kit; face-to-face support groups; tele-support groups; support and information hotline; website information; fact sheets; family and friends’ booklet; consumer information forums; online support forum; webinars/webcasts; and “other – please specify”.

Participants were also asked how likely they would be to use possible new support and information resources, with response options of “very likely”, “somewhat likely”, “not sure”, “somewhat unlikely”, and “very unlikely”. The resources were: a telephone-based wellbeing program that provides diet and exercise advice; an 8-week online program targeting anxiety and depression; an online support group; face-to-face information forums; a case management service; a factsheet of post treatment information for ovarian cancer survivors; a mobile app version of the OCA Resilience Kit (evidence-based information on living with ovarian cancer); a factsheet on the impact of ovarian cancer on sexuality; and a factsheet on managing fear of cancer recurrence.

In a separate item, participants were asked how likely they would be to use a fertility preservation decision aid (a tool to facilitate complex health decisions), with the same responses as for resources with the addition of “this service does not apply to me”.

Distributions of clinical characteristics and resource use were summarised using frequencies and proportions. The distributions of priority rankings were summarised using medians, interquartile ranges and ranges. Distributions were compared between those aged 19 to 49 (“younger participants”) and those aged 50 or above (“older participants”). This age division was chosen because reproductive stage may change life needs and disease progression, and the average age of menopause in Australia is 51 years [15]. Clinical characteristics and resource use were compared using χ^2^ tests. Priority rankings were compared using the Wilcoxon signed-rank test. Both general and OCA-specific resources were further classified as “technological” and “non-technological” and their use compared using using χ^2^ tests. Technological resources were those requiring the use of the telephone or internet; non-technological resources were those using paper or face-to-face contact. Data were analysed using Stata 15 (StataCorp. 2017. *Stata Statistical Software: Release 15*. College Station, TX: StataCorp LLC).

## Results

### Population demographics

The study cohort included participants aged 19 to 89 with the most common age bracket being 60-69 years of age (*n*=97, 33.7%, Table 1). Ninety-nine percent of participants identified as female (*n*=285). For reasons of brevity, this paper will refer to study participants as ‘women’ given that nearly all identified as female. Most participants reported living in a major city (*n*=215; 74.6%). There was no significant difference between the locality remoteness of younger respondents and older respondents (*p*=0.3). Fifty-seven percent of respondents (*n*=134) reported that they had been treated in a private hospital while 43% (*n*=101) were admitted to a public hospital for cancer treatment. Most respondents reported being diagnosed at Stage III (*n*=95; 45.5%) at time of diagnosis. Younger respondents (<50 years) were more likely to have been diagnosed with an early stage (Stage I and II) ovarian cancer (*n*=127; 60.8%) than older respondents (*n*=51; 32.3%; *p*<0.001).

**Table 1:** Demographics and clinical characteristics of participants who have had a diagnosis of ovarian cancer

	n (%)			
All Participants	288 (100.0)			
Age				
19-29 years	7 (2.4)			
30-39 years	22 (7.6)			
40-49 years	34 (11.8)			
50-59 years	80 (27.8)			
60-69 years	97 (33.7)			
70-89 years	48 (16.7)			
	Total, n (%)	Under 50 years old, n (%)	50 years old and over, n (%)	p-value^a^
Gender				
Female	285 (99.0)	63 (100.0)	222 (98.7)	0.4
Male	3 (1.0)	0 (0.0)	3 (1.3)	
Locality remoteness				
Major city	215 (74.6)	44 (69.8)	171 (76.0)	0.4
Inner regional	53 (18.4)	13 (20.6)	40 (17.8)	
Outer regional/ Remote	18 (6.3)	6 (9.5)	12 (5.3)	
Not specified	2 (0.7)	0 (0)	2 (0.9)	
Hospital care				
Private	134 (46.5)	29 (46.0)	105 (46.7)	0.2
Public	101 (35.1)	27 (42.9)	74 (32.9)	
Unsure/Unidentified	53 (18.4)	7 (11.1)	46 (20.4)	
Stage at time of diagnosis				
Stage I	46 (16.0)	19 (30.2)	27 (12.0)	0.002
Stage II	36 (12.5)	12 (19.0)	24 (10.7)	
Stage III	95 (33.0)	15 (23.8)	80 (35.6)	
Stage IV	32 (11.1)	5 (7.9)	27 (12.0)	
Unsure/Other	27 (9.4)	6 (9.5)	21 (9.3)	
Not specified	52 (18.1)	6 (9.5)	46 (20.4)	
Cancer pathway				
Primary treatment/ Recently diagnosed	18 (6.3)	8 (12.7)	10 (4.4)	0.09
Recovering from treatment	32 (11.1)	9 (14.3)	23 (10.2)	
Recurrence/ Palliative (EoL) care	46 (16.0)	9 (14.3)	37 (16.4)	
Remission	122 (42.4)	28 (44.4)	94 (41.8)	
Unsure/Other	18 (6.3)	3 (4.8)	15 (6.7)	
Not specified	52 (18.1)	6 (9.5)	46 (20.4)	
Time since first diagnosis				
<24 months	45 (15.6)	20 (31.7)	25 (11.1)	<0.001
24-59 months	94 (32.6)	21 (33.3)	73 (32.4)	
60-119 months	61 (21.2)	6 (9.5)	55 (24.4)	
120+ months	34 (11.8)	10 (15.9)	24 (10.7)	
Not specified	54 (18.6)	6 (9.5)	48 (21.3)	

^a^Chi-squared test (cell n≥5), Fisher’s exact test (cell *n*<5)

### Priorities

Just over half of the respondents (*n*=99, 51%) ranked fear of cancer recurrence as the most challenging aspect of living with ovarian cancer, followed by ‘feeling isolated’ (*n*=21, 11.9%, Table 2). The lowest priority was given to travelling costs. There were no significant differences to priority rankings by age.

**Table 2:** Ranking of challenges by participants who had or currently have a diagnosis of ovarian cancer (1, most challenging; 9, least challenging)

	Median (IQR)			p-value^a^
	Total (*n* = 288)	Under 50 years old (*n*=63)	50 years old and over (*n*=225)	
Fear of cancer recurrence	1 (1─4)	1 (1─3)	1 (1─4)	0.8
Feeling isolated	4 (2─7)	3.5 (2─6)	4 (2─7)	0.2
Financial concerns	5 (2─7)	3.5 (2─6)	5 (3─7)	0.1
Finding information on treatment options	5 (3─6)	5 (3─6)	5 (3─6)	0.8
Body image issues	5 (3─7)	5 (3─7)	5 (3─7)	0.2
Finding support for family and friends	6 (4─8)	5.5 (3─8)	6 (4─8)	0.08
Changes to sexual function	6 (4─8)	5.5 (3─7)	6 (4─8)	0.5
Finding information on genetic counselling	6 (4─8)	7 (5─8)	6 (4─8)	0.06
Travelling costs	7 (5─9)	8 (5─9)	7 (4─9)	0.2

^a^Wilcoxon signed-rank test for medians, chi-squared test for categorical variables

### Support types and resources accessed

When the participants were asked which supports and resources they used (Table 3), for most there were no differences by age. The vast majority (*n*=205; 71.2%) sought out support from friends and family, and this did not differ by age (*p*=0.05). Participants also often sought out support from their ‘hospital treatment team’ (*n*=149, 51.7%). OCA resources were also commonly accessed, with ‘information on the Ovarian Cancer Australia website’, referring to the general information available on the website, utilised by 48.3% (*n*= 139). Of the categorised OCA resources, the ‘resilience kit’ – a booklet produced by OCA with evidence-based information on living with ovarian cancer – was the most accessed (*n*=136, 47.2%). Supports and resources that were accessed differently across younger and older groups (respectively) were ‘information from other websites’ (47.6% vs 31.1%, *p*=0.02), ‘information sessions’ (9.5% vs 21.8%, *p*=0.03), ‘face-to-face support groups’ (6.4% vs 16.4%, *p*=0.04), and ‘webinars and webcasts’ (4.8% vs 16.9%, *p*=0.01). To determine whether the difference observed for use of ‘information on other websites’ (*p*=0.02) and ‘webinars and webcasts’ (*p*=0.01) was associated with age, we examined the relationship of age to use of any technological resource, which was not significant (*p*=0.2). To ascertain whether the difference observed for use of ‘information sessions’ (*p*=0.03) and ‘face-to-face support groups’ (*p*=0.04) was due to a preference for non-technological resources, we examined the relationship of age to the use of any non-technological resource, which was not significant (*p*=0.06).

**Table 3:** Resources accessed by people who have had a diagnosis of ovarian cancer

	n (%)			p-value^a^
	Total	Under 50 years old	50 years old and over	
Supports and resources accessed				
Friends and family	205 (71.2)	51 (81.0)	154 (68.4)	0.5
Cancer support group	85 (29.5)	15 (23.8)	70 (31.1)	0.3
Online forum/community	72 (25.0)	18 (28.6)	54 (24.0)	0.5
Telephone support service or group	40 (13.9)	10 (15.9)	30 (13.3)	0.6
One-on-one counselling	62 (21.5)	18 (28.6)	44 (19.6)	0.1
Hospital treatment team	149 (51.7)	32 (50.8)	117 (52.0)	0.9
Information on the OCA website	139 (48.3)	33 (52.4)	106 (47.1)	0.5
Information on other websites	100 (34.7)	30 (47.6)	70 (31.1)	0.02
Webinars or podcasts	38 (13.2)	5 (7.9)	33 (14.7)	0.2
Information sessions	55 (19.1)	6 (9.5)	49 (21.8)	0.03
Other hospital services (e.g. social work, psychology)	63 (21.9)	14 (22.2)	49 (21.8)	0.9
Other	41 (14.2)	7 (11.1)	34 (15.1)	0.4
Number of resources used				
0	52 (18.1)	6 (9.5)	46 (20.4)	0.1
1─3	89 (30.9)	24 (38.1)	65 (28.9)	
4+	147 (51.0)	33 (52.4)	114 (50.7)	
Median (IQR)	4 (2─5)	4 (2─5)	4 (1─5)	
Number of technological resources used				
0	106 (36.8)	18 (28.6)	88 (39.1)	0.3
1─2	126 (43.8)	30 (47.6)	96 (42.7)	
3+	56 (19.4)	15 (23.8)	41 (18.2)	
Median (IQR)	1 (0─2)	1 (0─2)	1 (0─2)	
Number of non-technological resources used				
0	58 (20.1)	7 (11.1)	51 (22.7)	0.02
1─2	123 (42.7)	36 (57.1)	87 (38.7)	
3+	107 (37.2)	20 (31.8)	87 (38.7)	
Median (IQR)	2 (1─3)	2 (1─3)	2 (1─3)	
OCA resources used				
Resilience kit	136 (47.2)	31 (49.2)	105 (46.7)	0.7
Face-to-face support groups	41 (14.2)	4 (6.4)	37 (16.4)	0.04
Tele-support	31 (10.8)	5 (7.9)	26 (11.6)	0.4
Support and information hotline	17 (5.9)	3 (4.8)	14 (6.2)	>0.9
Website information	103 (35.8)	27 (42.9)	76 (33.8)	0.2
Fact sheets	70 (24.3)	16 (25.4)	54 (24.0)	0.8
Family and friends' booklet	32 (11.1)	7 (11.1)	25 (11.1)	>0.9
Consumer information forums	27 (9.4)	27 (7.9)	22 (9.8)	0.7
Online support forum	25 (8.7)	7 (11.1)	18 (8.0)	0.4
Webinars and webcasts	41 (14.2)	3 (4.8)	38 (16.9)	0.01
Other	9 (3.1)	1 (1.6)	8 (3.6)	0.7
Number of OCA resources used				
0	134 (46.5)	29 (46.0)	105 (46.7)	0.9
1─2	54 (18.8)	13 (20.6)	41 (18.2)	
3+	100 (34.7)	21 (33.3)	79 (35.1)	
Median (IQR)	1 (0─3)	1 (0─3)	1 (0─4)	
Number of technological OCA resources used				
0	162 (56.3)	33 (52.4)	129 (57.3)	0.3
1─2	94 (32.6)	25 (39.7)	69 (30.7)	
3+	32 (11.1)	5 (7.9)	27 (12.0)	
Median (IQR)	0 (0─1)	0 (0─1)	0 (0─2)	
Number of non-technological OCA resources used				
0	139 (48.3)	30 (47.6)	109 (48.4)	0.8
1	61 (21.2)	15 (23.8)	46 (20.4)	
2+	88 (30.6)	18 (28.6)	70 (31.1)	
Median (IQR)	1 (0─2)	1 (0─2)	1 (0─2)	

^a^Chi-squared test (cell n≥5), Fisher’s exact test (cell *n*<5)

Just over fourteen percent of participants specified ‘other’ supports and resources used, including: support from GPs (*n*=7), support from groups on social media platforms (e.g. Facebook) (*n*=2) and massage therapy and meditation/mindfulness (*n*=3). Almost one in five respondents (*n*=52; 18.1%) accessed none of the supports or resources explored in the consumer survey; this proportion did not significantly differ by age (*p*=0.1).

### Likelihood of accessing resources

Echoing participant priority rankings, nearly two-thirds (*n*=137; 63.1%) were ‘very likely’ to access a ‘managing fear of cancer recurrence’ factsheet if available, 55.6% (*n*=120) would ‘very likely’ access a ‘post-treatment information’ factsheet, 29.9% (*n*=64), would be ‘very likely’ to use a ‘impact of ovarian cancer on sexuality’ factsheet, and 29.8% (*n*=65) would be ‘very likely’ to access a case management service (Table 4). These did not differ by age (*p*=0.8, 0.7, 0.05, and 0.9, respectively).

**Table 4:** Likelihood of people who have had a diagnosis of ovarian cancer accessing supports and resources

Likelihood of using …	N (%)			p-value*
	Total	Under 50 years old	50 years old and over	
… a telephone-based wellbeing program that provides diet and exercise advice (*n*=215)				
Very unlikely	36 (16.7)	10 (19.2)	26 (15.6)	0.9
Somewhat unlikely	30 (14.0)	8 (15.4)	22 (13.5)	
Not sure	34 (15.8)	7 (13.5)	27 (16.6)	
Somewhat likely	73 (34.0)	18 (34.6)	55 (33.7)	
Very likely	42 (19.5)	9 (17.3)	33 (20.3)	
… an 8-week online program targeting anxiety and depression (*n*=211)				
Very unlikely	27 (12.8)	3 (5.9)	24 (15.0)	0.3
Somewhat unlikely	31 (14.7)	6 (11.8)	25 (15.6)	
Not sure	26 (12.3)	5 (9.8)	21 (13.1)	
Somewhat likely	69 (32.7)	22 (43.1)	47 (29.4)	
Very likely	27 (12.8)	15 (29.4)	43 (26.9)	
… online support groups (*n*=207)				
Very unlikely	23 (11.1)	4 (8.0)	19 (12.1)	0.8
Somewhat unlikely	29 (14.0)	7 (14.0)	22 (14.0)	
Not sure	37 (17.9)	7 (14.0)	30 (19.1)	
Somewhat likely	78 (37.7)	20 (40.0)	58 (36.9)	
Very likely	40 (19.3)	12 (24.0)	28 (17.8)	
… face-to-face information forums (*n*=216)				
Very unlikely	17 (7.9)	3 (5.8)	14 (8.5)	0.4
Somewhat unlikely	24 (11.1)	6 (11.5)	18 (11.0)	
Not sure	41 (19.0)	14 (26.9)	27 (16.5)	
Somewhat likely	54 (25.0)	14 (26.9)	40 (24.4)	
Very likely	80 (37.0)	15 (28.9)	65 (39.6)	
… post-treatment information factsheet for ovarian cancer survivors (*n*=216)				
Very unlikely	10 (4.6)	1 (1.9)	9 (5.5)	0.7
Somewhat unlikely	4 (1.9)	1 (1.9)	3 (1.8)	
Not sure	17 (7.9)	4 (7.7)	13 (7.9)	
Somewhat likely	65 (30.1)	13 (25.0)	52 (31.7)	
Very likely	120 (55.6)	33 (63.5)	87 (53.1)	
… a mobile app version of the OCA resilience kit (*n*=210)				
Very unlikely	32 (15.2)	2 (3.9)	30 (18.9)	0.002
Somewhat unlikely	21 (10.0)	1 (2.0)	20 (12.6)	
Not sure	37 (17.6)	9 (17.7)	28 (17.6)	
Somewhat likely	56 (26.7)	16 (31.4)	40 (25.2)	
Very likely	64 (30.5)	23 (45.1)	41 (25.8)	
… factsheet on impact of OC on sexuality (*n*=214)				
Very unlikely	29 (13.6)	2 (3.9)	27 (16.7)	0.04
Somewhat unlikely	31 (14.5)	4 (7.7)	27 (16.7)	
Not sure	32 (15.0)	9 (17.3)	23 (14.2)	
Somewhat likely	58 (27.1)	17 (32.7)	41 (25.3)	
Very likely	64 (29.9)	20 (38.5)	44 (27.2)	
… factsheet on managing fear of recurrence (*n*=217)				
Very unlikely	11 (5.1)	1 (1.9)	10 (6.1)	0.8
Somewhat unlikely	5 (2.3)	1 (1.9)	4 (2.4)	
Not sure	14 (6.5)	4 (7.7)	10 (6.1)	
Somewhat likely	50 (23.0)	11 (21.2)	39 (23.6)	
Very likely	137 (63.1)	35 (67.3)	102 (61.8)	
… a fertility preservation decision aid (*n*=218)				
This service does not apply to me	171 (78.4)	31 (59.6)	140 (84.3)	<0.001
Very unlikely	9 (4.1)	2 (3.9)	7 (4.2)	
Somewhat unlikely	3 (1.4)	2 (3.9)	1 (0.6)	
Not sure	7 (3.2)	0	7 (4.2)	
Somewhat likely	11 (5.1)	4 (7.7)	7 (4.2)	
Very likely	17 (7.8)	13 (25.0)	4 (2.4)	
… a case management service (*n*=218)				
Very unlikely	36 (16.5)	7 (13.5)	29 (17.5)	0.9
Somewhat unlikely	21 (9.6)	6 (11.5)	15 (9.0)	
Not sure	42 (19.3)	10 (19.2)	32 (19.3)	
Somewhat likely	54 (24.8)	15 (28.9)	39 (23.5)	
Very likely	65 (29.8)	14 (26.9)	51 (30.7)	

^a^Chi-squared test (cell n≥5), Fisher’s exact test (cell *n*<5)

Age differences were seen across two resources. A greater proportion of younger respondents (*n*=23; 45.1%) were ‘very likely’ to use a mobile app version of the OCA resilience kit compared to older respondents (*n*=41, 25.8%; *p*=0.004). Also, a larger proportion of younger participants (*n*=13; 25%), compared to older participants (*n*=4; 2.4%), were ‘very likely’ to use a fertility preservation decision aid (*p*<0.001). Most participants (*n*=171, 78.4%; *p*=<0.001) responded that they would not use a fertility preservation decision aid as it did not apply to them. The response “This service does not apply to me” regarding the use of a fertility preservation aid, was selected by more older participants (*n*=140; 84.3%) than younger participants (*n*=31; 59.6%) upon age comparison analysis (*p*<0.001).

## Discussion

This consumer survey was conducted to analyse the support and information resources for ovarian cancer provided by OCA and in general. The current secondary analysis was intended to explore care experiences and priorities in support services based on feedback from women with ovarian cancer. Our main finding was that, in contrast to previous findings [16–18], age does not affect common concerns - fear of cancer recurrence, treatment side effects and the uncertainties surrounding ovarian cancer.

The main priority for study participants was to manage fear of cancer recurrence. In women with ovarian cancer, fear of cancer recurrence (and fear of cancer progression) can be overwhelming [19–22], and is more extreme than in women diagnosed with other common cancers [23]. Fear of cancer recurrence amongst women with breast cancer is considerably lower and a diagnosis of breast cancer does not elicit the same degree of stress about recurrence as a diagnosis of ovarian cancer does [24]. This is reasonable considering the average rate of recurrence in ovarian cancer is 70% [25], compared to 30% in breast cancer [26, 27] and under 5% in low-risk endometrial carcinoma (the most common gynaecological cancer [28]. Although we observe more cancer recurrence in later stage diagnoses of ovarian cancer, this is not to say that women diagnosed with earlier stages have less fear of cancer recurrence than those with late stage ovarian cancer [29]. Fear of cancer recurrence has been consistently associated with poorer quality of life, depression, anxiety and impaired daily functioning [30, 31]. The long-term effect of fear of cancer recurrence is exacerbated by the inability to come to terms with the priorities that people diagnosed with cancer experience [19, 29, 32]. Thus, there is need for support in managing this. Participants reported a high likelihood of using a factsheet for managing fear of cancer recurrence, an inexpensive option for support. For a psychological approach, a mind-body intervention (i.e. an intervention that aims to improve physical and mental health, e.g. yoga, tai chi, and Pilates) has shown to be effective for managing the fear of cancer recurrence/ progression in breast cancer patients [33, 34]. Although there have been no studies conducted to support this in ovarian cancer, this intervention has the potential to be adapted for women with ovarian cancer.

Differences by age - greater use of non-OCA websites and increased likelihood to use a mobile app amongst younger participants and older participants being more likely to use face-to-face support groups, information seminars and webinars – could, in part, be a reflection of confidence and comfort with technological resources [35] and also a reflection of lifestyle differences. Although the smart phone has increased technology access, it has been reported that older adults with chronic illness may not want to engage with internet-based and mobile health (mHealth) resources [36]. There is limited recent data in ovarian cancer, but our data suggest something similar. Although this technology-hesitancy may change over time as the aging population becomes more familiar with technology, it also illustrates that to ensure that patients access and gain benefit from resources they need to be designed with patient preferences in mind. In this case, having both online and paper-based resources may be useful.

Considering 78% of the group were over 50 years of age when they completed the survey (i.e. beyond their reproductive years), is it unsurprising that most (78%) participants indicated that the fertility decision aid was not of benefit to them. Most participants would have come to a point where they have no need for this information (having completed their families, have no desire for children, or may have already accessed fertility information). In addition to this, it came as no surprise that a greater proportion of younger women were interested in fertility decision. Fertility preservation is important to young women with ovarian cancer [7] and many younger women with ovarian cancer experience a significant amount of emotional distress when it comes to making a decision about fertility preservation [37]. Although there was only a small sub-set of participants that fit in this group (*n*=47), is it telling that four out of five of those who felt that a fertility decision aid would be relevant to them were also likely to want to use one. Fertility decision aids are effective tools to supporting oncofertility decision-making – with a growing body of evidence for improved decision-related outcomes in women with breast cancer, and other cancer types [38–40]. Clinical guidelines also recommend their use in the oncofertility context [41]. However, no decision aids exist for ovarian cancer. In considering the utility of a decision aid for this group, consideration needs to be given for the impact of poor prognosis (as families may need to take into account the possibility of a maternal loss on future children), higher risk of cancer recurrence after or during pregnancy, and reseeding risks from tissue that has not undergone cancer treatment. More research is still needed to explore and understand the nuances of the oncofertility decisions made by young women relating to fertility preservation in ovarian cancer.

### Limitations

The survey was subject to selection bias as we used a convenience sample recruited by advertisement on social media,. It is not possible to calculate a response rate, as there is no appropriate denominator for the sample – “reach” is an estimate of the number of people who were exposed to the advertisement, not those who engaged with it or could have been eligible to participate. It may be that the study sample is not representative of women with ovarian cancer in terms of age or priorities.

While the age of 50 was used as a cut-off to reflect change in reproductive status, potentially, a different cut off may have shown differences between groups. Further, the collection of age in categories limited comparisons by age. Relatively few younger women participated and their menopausal status was not recorded. We were thus unable to assess their priorities relating to the cessation of their reproductive life, if applicable. The survey also did not specify type of ovarian cancer. Another limitation to this survey was that the ‘usefulness’ of the resources which respondents accessed was not assessed. As noted elsewhere, no further detail was obtained when respondents selected “This service does not apply to me” regarding their likelihood of using a fertility preservation decision aid.

## Conclusion

Supportive care priorities for women with ovarian cancer did not vary by age. Fear of recurrence was the primary concern. Future research should prioritise the development of interventions to address these priorities to improve patient experiences and their quality of life. Modes of information delivery need to consider age of the target audience –with younger women preferring to interact with technology and being more eager to receive information via mobile-app type interventions and tools compared to older women. Fertility is more important to younger women and a fertility preservation decision aid may address this need.

## Data Availability

Data are available on request at the discretion of Ovarian Cancer Australia.

## Code availability

N/A.
